# Associations of Bone Mineral Density with Lean Mass, Fat Mass, and Dietary Patterns in Postmenopausal Chinese Women: A 2-Year Prospective Study

**DOI:** 10.1371/journal.pone.0137097

**Published:** 2015-09-03

**Authors:** Yongjie Chen, Jing Xiang, Zhiqiang Wang, Yaming Xiao, Dongmei Zhang, Xia Chen, Huiting Li, Meina Liu, Qiuju Zhang

**Affiliations:** 1 Department of Biostatistics, Public Health College, Harbin Medical University, Harbin City, Heilongjiang Province, China; 2 School of Medicine, University of Queensland, Royal Brisbane & Women's Hospital, Brisbane, Queensland, Australia; 3 The First People’s Hospital of Tengzhou, Zaozhuang City, Shandong Province, China; 4 Department of Epidemiology and Evidence-based Medicine, Public Health College, Changsha Medical University, Changsha City, Hunan Province, China; 5 The Harbin Institute of Technology Hospital, Harbin City, Heilongjiang Province, China; Oklahoma State University, UNITED STATES

## Abstract

**Objective:**

To assess factors associated with bone mineral density (BMD) in postmenopausal women in a longitudinal study, and to examine the relative contribution of lean mass, fat mass, dietary patterns, and years since menopause to BMD.

**Methods:**

Two hundred and eighty-two postmenopausal women were randomly selected from Hongqi Community Health Center, in Harbin City, China. All participants were followed up from 2009 to 2011. Dietary data were collected using a Food Frequency Questionnaire. BMD of the left hip, the lumbar spine, and the total body, and the body composition were measured by dual-energy X-ray absorptiometry at baseline and follow-up.

**Results:**

Lean mass and fat mass were positively associated with BMD of the spine, hip, and the total body at both baseline and follow-up. The association between fat mass and BMD at the spine at baseline (*P* = 0.210) and at the spine (*P* = 0.116) and hip (*P* = 0.073) in the second year was not statistically significant when height was adjusted. Six dietary patterns were identified but only cereal grains-fruits pattern (*P* = 0.001 in the spine, *P* = 0.037 in hip) and milk-root vegetables pattern (*P* = 0.010 in hip) were associated with BMD of the spine and hip. The linear mixed model of follow-up data showed that lean mass, years since menopause, and age of menophania were the significant determinants of BMD of all sites. Moreover, lean mass was the best determinant of BMD (VIP = 1.936).

**Conclusion:**

Lean mass, years since menopause, age of menophania and dietary patterns are the important determinants of BMD of the spine, hip, and the total body. Lean mass is the best determinant of BMD.

## Introduction

Osteoporosis is a major public health problem all over the world for its high morbidity among the aging populations especially for women[1]. Osteoporotic fracture is the most serious outcome of osteoporosis and commonly results in permanent disability, admission to institutional care, and even death[2–5]. Low bone mineral density (BMD) is one of the main pathogenic factors of osteoporotic fracture[6, 7]. BMD has been widely accepted as a surrogate measure for the diagnosis of osteopenia and osteoporosis[8, 9]. Therefore, improved understanding of the primary predictors of BMD would have important implications for public health and preventive medicine.

A large number of cross-sectional studies have found a significant relationship between BMD and age, body weight, and body composition in postmenopausal women [10–13]. Numerous other physiologic, health, and lifestyle factors, such as years since menopause, history of fracture, smoking status, alcohol consumption, blood pressure, dietary calcium intake, estrogen replacement therapy, use of steroid drugs, muscle strength, past and current physical activity level, dependence on assistance for instrumental activities of daily living (IADL), gait and balance impairment, and social (educational attainment and family income) and psychophysical health status are also associated with BMD in the postmenopausal women[5, 14–17]. However, it has been shown that longitudinal studies can provide a better understanding of the contribution of these factors to BMD[6, 18, 19]. Because only a few prospective studies investigating the influencing factors associated with bone loss over a long-term of postmenopausal life have been reported, limited information is available on whether body weight, body composition, and other physiologic, health, and lifestyle factors have a direct effect on the rate of BMD change in elderly women. Furthermore, none of the previous studies have taken a full consideration of dietary pattern of participants. Different dietary patterns have different characteristics of nutrient intake, such as calcium element, vitamin D, and dietary fiber (DF). These nutrients considerably affect the concentration and absorption of calcium element in body. On the other hand, although Ho-Pham[20], Chen Z[21], and Gnudi S[22]reported the relative contribution of lean mass and fat mass to BMD, this relative contribution has been highly contentious. A meta-analysis by Ho-Pham reported that the correlation between lean mass and BMD was significantly higher than the correlation between fat mass and BMD in women[23]. Although the effects of lean mass and fat mass on BMD were comparable in postmenopausal women, the correlation for lean mass was still greater than correlation for fat mass. So far, however, the relative importance of other physiologic, health, and lifestyle factors to BMD is still unknown. Similar studies in Chinese population[1, 24, 25] were of only cross-sectional designs. In this paper, a 2-year prospective study was designed to investigate the relative contribution of the primary factors, including dietary patterns, years since menopause and other physiologic factors, in addition to body composition, to BMD in Chinese women.

Considering the effect of other primary factors simultaneously, we hypothesized that lean mass was the key determinant of BMD, and dietary patterns was also an important predictor of BMD. Therefore, the aims of this study were: (i) to identify the dynamic predictors of BMD over a 2-year period; (ii) to assess the relative contribution of lean mass, fat mass, dietary patterns, and years since menopause to BMD over the follow-up period in postmenopausal Chinese women.

## Methods

### Subjects and Follow-Up

In Harbin City, China, Hongqi Community Health Center was randomly selected as the target community from all teaching community hospitals of Harbin Medical University. In this community, 373 volunteers were randomly recruited and those who met the following inclusion criteria were included in the study. According to a previous study[26], the postmenopausal status was defined as menopause at least one year since the last natural menstruation. The postmenopausal women aged between 50 and 65 years at the time of the study and having lived in Harbin for at least 5 years were eligible for this study. Subjects were excluded if they: (i) had disorders of calcium metabolism or calcium absorption; (ii) had disorders of bone; (iii) had gastrointestinal disease, coronary heart disease, stroke, diabetes, cancer, thyroid or parathyroid disease, chronic liver disease or chronic kidney disease; (iv) suffered from ovarian surgery, premenopausal hysterectomy, gastric resection and thyroidectomy; (v) used estrogen at the time of the study or had taken drugs for a month or more; and (vi) were likely to migrate. Finally, 282 women were included in the study. The study protocol was approved by the National Institute of Nutrition and Food Safety Chinese Center for Disease Control and Prevention Ethical Review Committee. The individual in this manuscript has given written informed consent to publish these case details.

During the two year follow-up period, we visited subjects at baseline, and then one and two years after the baseline to collect data on demographic and socioeconomic background, body composition, bone mineral density, food frequency, blood specimens of morning fasting and urine of 24 hours. Data of food frequency were collected using the Food Frequency Questionnaire (FFQ), which was designed in case report form.

### Structured Questionnaire

The structured questionnaire included the following items: demographic and socioeconomic background, age at menopause, estrogen replacement therapy, past medical history, food frequency, past physical activity level, reproductive and menstrual history, and mental status. The validity and reliability of the questionnaire was tested. Participants were face-to-face interviewed by trained investigators using the validated questionnaire. Each subject recalled the diet of the past year with the help of estimating portion size through detailed pictorial information. The dietary nutrient intake was estimated using the 3-day food records, such as energy, protein, calcium, vitamin D, and other microelements.

Physical measurements were obtained according to standardized protocol. Height was measured without shoes to the nearest 0.1cm, and weight with only light clothing to the nearest 0.1kg. The means of three repeated measures were calculated and recorded. Body mass index (BMI) was calculated as weight (in kg) divided by height (in m) square. Waist circumference was measured at the minimum circumference between the iliac crest and the rib cage while hip circumference was measured at the maximum protuberance of the buttocks. Waist and hip circumferences were used to calculate the waist-hip-ratio (WHR).

### Body Composition and Bone Mineral Density Measurements

Baseline and follow-up BMD values of left hip (femoral neck, trochanter, Ward’s triangle), lumbar spine (L2-L4), and total body, and body composition were measured by dual-energy X-ray absorptiometry (DXA, Norland Corp, USA) in Harbin Orthopedics Hospital. Body composition was represented by lean mass (bone mineral excluded) and fat mass (in kg). The long-term precision values, expressed as coefficient of variation (CV) for repeated measurements over two years, were 1.0% for total body, 1.2% for hip, and 1.0% for lumbar spine.

### Dietary Assessment and Dietary Patterns Derivation

At baseline, dietary nutrients intake was assessed using a validated FFQ developed in a population survey[27]. The FFQ consisted of 80 food items. Each subject recalled the frequency and the usual amount of consumption of each food item over the past year. For seasonally consumed vegetables and fruits, subjects were further asked about the duration of such food consumption over the past year.

To determine the dietary patterns, individual food items were first aggregated into groups. A total of thirteen separate food groups were formed based on the similarity of food types and nutrient composition. The percentage contribution to the total energy from each food group was calculated as the energy intake from that food group divided by the total energy intake and multiplying by 100. Factor analysis was conducted with varimax rotation using the thirteen food groups. Factors were retained with eigenvalues greater than 1.0, a scree plot and interpretability. Finally, six dietary patterns were identified.

### Variables Importance to BMD

To better understand the relative importance of all factors to BMD, variable importance (VIP) to BMD was produced in this study. When 1) there are only a small number of factors, 2) those factors are not significantly redundant (collinear), and 3) a well-understood relationship to the response, multiple linear regression (MLR) can be a good way to assess the predictive values of those factors. However, if any of these three conditions breaks down, MLR can be inefficient or inappropriate[28]. Given this condition, partial least square (PLS) developed in the 1960’s by Herman Wold was used. PLS is widely used to construct predictive models to solve the highly collinear when the sample size was small but the variables can number in the hundreds even thousands and are likely to be highly collinear[29]. Considering the sample size and the hundreds of factors in this study, PLS was used to obtain VIP to BMD.

### Statistical Analysis

The analysis was conducted in four steps. First, dietary patterns were derived by factor analysis. Second, VIPs to BMD at all sites were estimated using PLS for factors including demographic and socioeconomic background, body composition, dietary patterns, past medical history, past physical activity level, and years since menopause. Third, the relationships between BMD and the primary variables such as lean mass, fat mass, height, and years since menopause at the baseline and follow-up were assessed. Pearson and Spearman correlation were used to analyze the association between BMD and the primary factors. Partial correlation was used to assess the strength of association adjusting for height and calcium supplementation. Finally, for follow-up data, linear mixed model was used to analyze the association between BMD and the primary variables with greater VIP to BMD. Three BMD measurements in each site were taken as dependent variables. The spine, hip and total body values were used to structure three linear mixed models, respectively. Lean mass and fat mass of three measurements were taken as time specific independent variables. And other variables, including age, years since menopause, dietary patterns, height, weight, systolic blood pressure (SBP),WHR, change of weight since menopause, age of menophania, educational attainment, occupation, family income and physical activity level, were taken as covariates. The procedure of PROC MIXED was used to estimate the parameters of linear mixed model. Since this study was based on a randomized controlled trial, all analyses were adjusted for calcium supplementation.

Basic characteristics of subjects were reported as mean and SDs for continuous variables and numbers and percentages for categorical variables. All analyses were conducted using the programs of SAS v9.2 (SAS Institute, Inc., Cary, NC, USA). All tests were two-tailed with a 5% level as the level of significance.

## Results

### Descriptive Variables

The mean age of all subjects was 56.1 (SD = 3.8) years and the mean time since menopause was 6.7 (SD = 4.4) years. Table 1 provides characteristics of all subjects at baseline and follow-up and body composition and bone measurements. During the follow-up, 212 subjects participated in the first year and 202 in the second year assessments, and the drop-out rates were 24.8% and 28.4%, respectively.

**Table 1:** The characteristics of participants at baseline and follow-up.

Factors	Category	Mean/Frequency	SDs/Percent
Age (year)		56.11	3.83
Age of menophania (year)		15.70	1.88
Years since menopause (year)		6.70	4.40
WHR^*^		0.85	0.05
Height (cm)		158.06	5.30
Weight(kg)		61.84	8.40
Change of weight since menopause (kg)		2.11	5.44
SBP^#^ (mmHg)		128.24	18.72
Spine^1st^ BMD (g/cm^2^)		0.90	0.16
Spine^2nd^ BMD (g/cm^2^)		0.92	0.16
Spine^3rd^ BMD (g/cm^2^)		0.90	0.15
Hip^1st^ BMD (g/cm^2^)		0.55	0.11
Hip ^2nd^ BMD (g/cm^2^)		0.57	0.11
Hip^3rd^ BMD (g/cm^2^)		0.58	0.12
Total body^1st^ BMD (g/cm^2^)		0.91	0.09
Total body ^2nd^ BMD (g/cm^2^)		0.92	0.12
Total body ^3rd^ BMD (g/cm^2^)		0.92	0.08
Lean mass^1st^ (kg)		37.13	4.90
Lean mass^2nd^ (kg)		35.95	4.67
Lean mass^3rd^ (kg)		34.63	4.60
Fat mass^1st^ (kg)		21.90	4.71
Fat mass^2nd^ (kg)		24.08	5.34
Fat mass^3rd^ (kg)		24.28	5.20
Educational attainment			
	Below junior middle school	22	7.80
	Junior middle school	125	44.33
	Senior middle school	88	31.20
	University or above	47	16.67
Occupation			
	Administration	57	20.21
	Clerical work	79	28.01
	Laborer	106	37.59
	Other	40	14.19
Family income			
	< = 1000¥	69	24.47
	1001~1500¥	84	29.79
	1501~2000¥	46	16.31
	2001~3000¥	40	14.18
	>3000¥	43	15.25
Physical activity level			
	Little	53	18.79
	Median	94	33.33
	Many	74	26.25
	More	61	21.63

^*^waist-hip-ratio

^#^systolic blood pressure.

### VIP to BMD

Demographic and socioeconomic characteristics, body composition, dietary patterns, past medical history, past physical activity level, and years since menopause were analyzed using PLS to obtain the VIP to BMD. These analyses were conducted with BMD of the spine, hip, and the total body from the follow-up data as the dependent variables. The first sixteen VIPs to BMD were in the following order: lean mass, weight, years since menopause, height, age, fat mass, family income, educational attainment, dietary pattern 2 (frequent intake of rice, cooked wheaten food, fried food, and other grains and little intake of fruits), occupation, age of menophania, dietary pattern 3 (frequent intake of milk and little intake of root vegetables), physical activity level, WHR, systolic blood pressure (SBP), and change of weight since menopause.

### Contribution of Lean Mass and Fat Mass to BMD: Cross-Sectional Analyses

Table 2 provides correlation coefficients among single body composition, height, and years since menopause and BMD of the spine, hip, and the total body at baseline and follow-up adjusting for calcium supplementation. The lean mass and fat mass were the significant determinants of BMD at all sites at baseline and follow-up. Moreover, the correlation coefficients of lean mass with BMD at any site were greater than those of fat mass at both baseline and follow-up. Height, as a body size measurement, was positively correlated with BMD at all sites at baseline. The variable of years since menopause was inversely associated with the baseline BMD at all sites.

**Table 2:** Correlation of BMD of the spine, hip, and the total body with body composition at baseline and follow-up (adjusting for calcium supplementation).

Parameters	BMD					
	Spine		Hip		Total body	
	*r*	*P*	*r*	*P*	*r*	*P*
**The baseline**						
Lean mass	0.3726	<.0001	0.4527	<.0001	0.4569	<.0001
Fat mass	0.1354	0.0230	0.2156	0.0003	0.2235	0.0002
Height	0.3181	<.0001	0.3629	<.0001	0.3885	<.0001
Years since menopause	-0.3711	<.0001	-0.2940	<.0001	-0.3626	<.0001
**The first year**						
Lean mass	0.3068	<.0001	0.4105	<.0001	0.4720	<.0001
Fat mass	0.2524	0.0003	0.3003	<.0001	0.3578	<.0001
**The second year**						
Lean mass	0.3522	<.0001	0.4239	<.0001	0.4648	<.0001
Fat mass	0.1712	0.0156	0.2025	0.0040	0.2832	<.0001

Table 3 shows partial correlation coefficients between the body composition and BMD of the spine, hip, and the total body adjusting for height and calcium supplementation at baseline and follow-up. Lean mass was positively correlated with BMD at all sites at both baseline and follow-up. However, the association of fat mass with BMD at the spine at baseline was not statistically significant, neither at the spine and hip in the second year.

**Table 3:** Partial correlation of BMD of the spine, hip, and the total body with body composition at baseline and follow-up (adjusting for height and calcium supplementation).

Parameters	BMD					
	Spine		Hip		Total body	
	*r*	*P*	*r*	*P*	*r*	*P*
**The baseline**						
Lean mass	0.2500	<.0001	0.3226	<.0001	0.3129	<.0001
Fat mass	0.0750	0.2103	0.1604	0.0071	0.1617	0.0066
**The first year**						
Lean mass	0.1825	0.0092	0.3170	<.0001	0.3519	<.0001
Fat mass	0.1834	0.0088	0.2305	0.0009	0.2821	<.0001
**The second year**						
Lean mass	0.2496	0.0004	0.2938	<.0001	0.3332	<.0001
Fat mass	0.1119	0.1164	0.1273	0.0732	0.2093	0.0030

### Linear Mixed Model of the Primary Variables on the BMD: 2-Year Follow-Up Data

Table 4 shows the coefficients of linear mixed model when only lean mass and fat mass were entered into the model as the predictors of BMD. Lean mass was a significant predictor of BMD of the spine, hip, and the total body (*P*<0.0001). Fat mass was a significant determinant of BMD in the total body (*P*<0.0001) but not in the spine (*P* = 0.075) and hip (*P* = 0.124).

**Table 4:** The results from linear mixed model of lean mass and fat mass on BMD of the spine, hip, and the total body: 2-year follow-up data.

Body composition	BMD					
	Spine		Hip		Total body	
	*β*	*P*	*β*	*P*	*β*	*P*
Lean mass	0.0105	<.0001	0.0069	<.0001	0.0038	<.0001
Fat mass	0.0022	0.0753	0.0014	0.1240	0.0032	<.0001

Table 5 shows the coefficients of linear mixed model of multiple factors on the BMD at all sites. Lean mass, years since menopause and age of menophania were significant determinants of BMD at all sites over the 2-year study period. Years since menopause was a significant risk factor of BMD of the spine (*P*<0.0001), hip (*P* = 0.0002) and the total body (*P*<0.0001). Weight was positively correlated with BMD of the spine and total body (*P* = 0.042 and *P*<0.0001, respectively) and was borderline significance in hip (*P* = 0.069). Dietary pattern 2 was a significant influencing factor of BMD of the spine (*P* = 0.001) and hip (*P* = 0.037). Dietary pattern 3 was a significant predictor of BMD of hip (*P* = 0.010). However, neither dietary pattern 2 nor dietary pattern 3 was a significant determinant of BMD of the total body. On the other hand, family income and physical activity level had significant relationships with BMD of hip (*P* = 0.003 and *P* = 0.042, respectively). The significant associations of SBP with BMD of the spine and fat mass and height with BMD of the total body were also found. WHR was negatively related to BMD of the spine and hip (*P* = 0.008 and *P* = 0.011, respectively). In contrast, no significant relationship was observed between BMD of all sites and the following baseline characteristics: age, change of weight since menopause, educational attainment, and occupation.

**Table 5:** The results from linear mixed model of the primary variables on BMD of the spine, hip, and the total body: 2-year follow-up data.

Factors	BMD					
	Spine		Hip		Total body	
	*β*	*P*	*β*	*P*	*β*	*P*
Lean mass	0.0056	0.0228	0.0045	0.0188	0.0007	<.0001
Fat mass	-0.0017	0.4570	-0.0013	0.4557	0.0013	<.0001
Age	-0.0008	0.7068	-0.0006	0.7028	0.0021	0.2145
Years since menopause	-0.0087	<.0001	-0.0054	0.0002	-0.0060	<.0001
Dietary pattern 2	-0.0189	0.0006	-0.0087	0.0368	-0.0075	0.0783
Dietary pattern 3	0.0054	0.3261	0.0108	0.0104	0.0005	0.9017
Height	0.0007	0.5948	-0.0007	0.5247	0.0021	0.0414
Weight	0.0046	0.0420	0.0031	0.0689	0.0037	<.0001
SBP^*^	0.0007	0.0266	-0.0001	0.5585	-0.0002	0.3943
WHR^#^	-0.3577	0.0075	-0.2593	0.0110	-0.1146	0.2613
Change of weight since menopause	-0.0010	0.3348	0.0006	0.4358	-0.0008	0.3436
Age of menophania	-0.0093	0.0011	-0.0057	0.0090	-0.0055	0.0148
Educational attainment	0.0135	0.0747	-0.0004	0.9523	-0.0083	0.1659
Occupation	-0.0011	0.8599	-0.0015	0.7606	-0.0019	0.7094
Family income	0.0069	0.1069	0.0098	0.0029	0.0063	0.0574
Physical activity level	0.0078	0.1390	-0.0083	0.0419	0.0065	0.1123

^*^systolic blood pressure

^#^waist-hip-ratio

## Discussion

Although many studies have been reported correlations between body composition and BMD, the relative contribution of lean mass and fat mass to BMD in postmenopausal women is a contentious issue. In this prospective study, the relative contribution of body composition measurements and other factors to BMD was examined. To our knowledge, this is the first longitudinal study to investigate the relative contribution of lean mass, fat mass, and dietary patterns to BMD in the postmenopausal Chinese women, whose body composition and lifestyle are possibly different from western populations[30]. Previous cross-sectional studies showed that both lean mass and fat mass were positively correlated with BMD of the spine, hip, and the total body. When adjusting for height, partial correlation coefficients showed that the association of fat mass with BMD at the spine at baseline and at the spine and hip in the second year was eliminated. The VIP of lean mass to BMD are prior to the VIP of fat mass in the PLS analysis. Therefore, lean mass was a better determinant of BMD than fat mass. The results from the multiple factors linear mixed model demonstrated that lean mass, not fat mass, was the significant predictor of BMD. Although some previous studies reported the same conclusions[11, 18, 20, 25, 31, 32], only the correlation between BMD and body composition adjusting for body size was analyzed in those studies. The effects of other factors such as years since menopause, dietary patterns, past physical activity level, and demographic and socioeconomic background on BMD were not considered simultaneously. The relative contribution of these factors to BMD was unknown. The present paper used PLS to obtain VIP of all variables to BMD. Then the top sixteen factors entered into the linear mixed model of the BMD follow-up data. Therefore, this paper provided a more comprehensive and convincing evidence on relative contribution of body composition and other factors to the determination of BMD.

In this study, dietary pattern of the participants, in addition to dietary calcium intake, was considered. Factor analysis identified six dietary patterns and 64.61% variance was explained. Since the FFQ was filled using the diet data of participants of the past year, recall bias was inevitable. The imperfect data might lead to the poor dietary pattern and lesser variance to be explained. Therefore, only dietary pattern 2 and dietary pattern 3 were entered into the top sixteen of all variables in the PLS analysis. Dietary pattern 2 was characterized by a frequent intake of rice, cooked wheaten food, fried food, and other grains and little intake of fruits. It was negatively correlated with BMD of the spine and hip. Dietary pattern 3 was dominated by a frequent intake of milk and little intake of root vegetables and was positively correlated with BMD of hip. This demonstrated that milk supplementation might have beneficial effect on BMD, which has been demonstrated by many randomized controlled trials[33–35].

From the results of linear mixed model, years since menopause was negatively associated with BMD of all sites since estrogen will obviously decrease after menopause, and estrogen is known to inhibit bone resorption by inducing osteoclasts apoptosis[36]. Menopause is accompanied by dramatic body composition changes, including an increase in total body and central adiposity, decrease in gynoid fat proportion, and a significant decrease in total and regional BMD[37, 38]. Therefore, postmenopausal women with more years since menopause were more likely to experience rapid bone loss and develop osteoporosis.

WHR, as a marker for visceral fat, was negatively associated with BMD of the spine and hip in the multiple factors linear mixed model. Therefore, fat distribution might have different effects on BMD of different sites. This finding confirmed the conclusion by Zillikens MC[39].

In the literature, BMI is often used as body size as a potential confounder when assessing the association between a factor and BMD. However, because of lack of empirical evidence and difficulty in interpretation, this usage is sub-optimal. Empirically, weight and height are better agency of body size than BMI[20]. In this study, height, rather than weight, was chosen as an agency of body size, because the correlation between fat mass and height (*r* = 0.236) was lower than the correlation between fat mass and weight (*r* = 0.828).

Although a few longitudinal studies investigating the influencing factors of BMD have been reported, most of them only focused on the correlation between the changes of lean mass and fat mass and the changes of BMD over time. Since these studies did not make full use of the original data of longitudinal study, the dynamic relationship between body composition and BMD could not be completely assessed. In addition, these studies only investigated the association between body composition and BMD, without adjusting for the effect of other factors, such as general conditions, physiologic factors and lifestyle factors. The strength of this paper was apparent. PLS was used to variables selection for all variables and top sixteen factors entered into the linear mixed model for the follow-up data. BMD of three time measurements were taken as the dependent variables, lean mass and fat mass as the time specific independent variables and the rest as the covariates. Therefore, the findings of this study would improve our understanding of the dynamic contributions of lean mass, fat mass, and other factors to BMD in a longitudinal study.

Limitations to the present study should be noted. Firstly, the study subjects were selected from one clinic, and they were unlikely to represent the general population. Secondly, the subjects were all Chinese. The results might not be generalized to other ethnicities. Thirdly, this study was based on a randomized controlled trial. Although the calcium supplementation was adjusted for in all analyses, it is possible that the influence was not completely eliminated.

In conclusion, this longitudinal study suggests that lean mass, not fat mass, age of menophania, and years since menopause are the determinants of BMD of the spine, hip, and the total body. More importantly, lean mass is the best predictor of BMD. The dietary pattern (cereal grains-fruits pattern) will accelerate bone loss in the spine and hip, and the dietary pattern (milk-root vegetables pattern) will improve the BMD of hip.
